# *Clostridium leptum* group bacteria abundance and diversity in the fecal microbiota of patients with inflammatory bowel disease: a case–control study in India

**DOI:** 10.1186/1471-230X-13-20

**Published:** 2013-01-26

**Authors:** Jayakanthan Kabeerdoss, Vijayalakshmi Sankaran, Srinivasan Pugazhendhi, Balakrishnan S Ramakrishna

**Affiliations:** 1 The Wellcome Trust Research Laboratory, Department of Gastrointestinal Sciences, Christian Medical College, Vellore, 632004, India

**Keywords:** Clostridial cluster IV, Dysbiosis, Crohn’s disease, Ulcerative colitis, Short chain fatty acids

## Abstract

**Background:**

Alterations in the fecal bacterial flora occur in inflammatory bowel disease (IBD). We examined the abundance and diversity of *Clostridium leptum* group, an important group of carbohydrate-fermenting bacteria, in the feces of patients with IBD and compared them with healthy controls.

**Methods:**

Seventeen healthy controls (HC), 20 patients with Crohn’s disease (CD) and 22 patients with ulcerative colitis (UC) participated in the study. DNA extracted from fecal samples was amplified by PCR targeting 16S rRNA gene sequences specific to *C. leptum* group. The PCR product was subjected to temporal temperature gradient electrophoresis (TTGE) and the number and position of individual bands were noted and diversity was estimated. The identity of bands at different positions was confirmed by cloning and sequencing. Real time quantitative PCR with Mesa Green, targeted at specific 16S rRNA gene sequences, was used to quantitate *C. leptum* group and its most prominent constituent, *Faecalibacterium prausnitzii*.

**Results:**

Twenty five different operational taxonomic units (OTUs, equivalent to species) were identified constituting the *C. leptum* group in these participants. Their sequences were deposited in GenBank [accession numbers GQ465348 to GQ465370]. OTU number was significantly reduced in CD (7.7±3.7, mean±SD) and UC (9.0±3.0) compared to HC (11.9±2.2) (P=0.0005). The Simpson D index of alpha diversity was not significantly different between the three groups. Total numbers of *C. leptum* group bacteria and *F. prausnitzii* were reduced in both CD and UC compared to HC (P=0.0036 and P<0.0001 respectively). Disease activity did not influence numbers of *C. leptum* or *F. prausnitzii* in patients with CD or UC.

**Conclusion:**

*C. leptum* numbers and diversity were significantly reduced in both CD and UC suggesting that alterations noted were not specific to one disease. This could contribute to reduced short chain fatty acid production in IBD.

## Background

The *Clostridium leptum* group of bacteria is one of the dominant groups of fecal bacteria in adult humans, constituting 16-25% of the fecal microbiota [1,2]. This group, also called Clostridial cluster IV, includes *Faecalibacterium prausnitzii* and certain species of *Eubacterium* and *Ruminococcus*[3]. Members of this group synergize with other intestinal microbiota to ferment unabsorbed dietary carbohydrate, producing short chain fatty acids of which butyrate is the major energy source for the colonic epithelium and profoundly influences intestinal epithelial function. *F. prausnitzii*, the most abundant member of this group, is a major producer of butyrate through carbohydrate fermentation [4]. It has acquired special status because of its association with anti-inflammatory effects in the gastrointestinal tract [5]. Any alteration in the composition of the gut microbiota that changes the microbial balance is likely to have repercussions on human health.

Inflammatory bowel diseases (IBD), comprising the two diseases ulcerative colitis (UC) and Crohn’s disease (CD) are thought to result from disordered innate immune reactions to luminal microbiota in individuals with the appropriate genetic disposition [6]. A number of studies indicate that microbiota alterations, termed dysbiosis, are common in IBD [7-14]. These alterations include reduction of total microbial diversity, increased numbers of *Enterobacteriaceae*, increased abundance of *Bacteroides-Prevotella*, increase or decrease in bifidobacteria, and decrease in the numbers of Firmicutes (especially *Clostridium coccoides-Eubacterium rectale* and *F. prausnitzii*). Most of the changes described have been in patients with active CD. An earlier study suggested that *F. prausnitzii* numbers were decreased in active CD but increased in active UC [8]. It is not known whether these changes are specific to CD, or whether they reflect changes that are more universally present in patients with IBD. It is also not known whether these changes in the microbiota persist in quiescent IBD.

In the present study we investigated the species diversity of the *C. leptum* group of bacteria in the feces of healthy individuals and patients with IBD in remission using amplification of the 16S rRNA gene followed by temperature gradient electrophoresis. We also quantitated *C. leptum* group and *F. prausnitzii* using real time PCR targeted at the 16S rRNA gene sequences specific to these bacterial communities.

## Methods

### Participants

Seventeen healthy control (HC) volunteers, 20 patients with CD and 22 patients with UC participated in this study. Participants were identified in the IBD clinic of the department, informed about the purpose of the study, and provided written consent. CD and UC were diagnosed on the basis of Asian consensus criteria for diagnosis of these diseases [15]. Disease activity status was assessed by the Crohn’s disease activity index and Truelove-Witts scoring system respectively [16,17]. Controls were identified from apparently healthy individuals visiting the general medical clinics of the hospital for routine health checkup. Participants who had taken antibiotics within the last three months were excluded. Fresh samples of feces were collected and transported within three hours to the laboratory, where they were stored in aliquots at -20°C for processing in batches. The protocol was approved by the institutional ethics committee of the Christian Medical College, Vellore.

### DNA extraction and polymerase chain reaction amplification

Total DNA was extracted from ~250 mg (wet weight) of fecal sample using the QIAamp DNA stool mini kit (Qiagen, Hilden, Germany) as per manufacturer’s instructions. The DNA was eluted in a final volume of 200 μL and stored at -20°C. The concentration and integrity of the nucleic acids were determined by electrophoresis on 1% agarose gel containing ethidium bromide. *C. leptum* group DNA sequences were amplified using primers that targeted sequences in the 16S rRNA gene specific to that group. The primers that were used were sg-Clept-F (Forward 5^′^-GCACAAGCAGTGGAGT-3^′^) and Clept-GC-R3 (Reverse with GC Clamp 5^′^-CTTCCTCCGTTTTGTCA-3^′^) [18]. Polymerase chain reaction (PCR) was performed in a reaction volume of 20 μl containing 1X PCR buffer, 2.5 mM MgCl_2_, 200 mM each dNTP, 250 nmol of both forward and reverse primers, 2.5 U of Jumpstart Taq polymerase (Sigma Aldrich Co, St. Louis, MO, USA), and approximately 10 ng of DNA template. Samples were amplified in a MJ Mini thermal cycler (Bio-Rad, Hercules, CA, USA) using the following program: 95°C for 1 minutes, 30 cycles of 95°C for 30 seconds, 56°C for 30 seconds, 72°C for 30 seconds, and finally 72°C for 10 minutes. PCR products were analyzed by electrophoresis on 2% agarose gel containing ethidium bromide to check their size (239 bp) and estimate their concentration.

### Temporal temperature gradient electrophoresis

We subjected the PCR product to temporal temperature gradient electrophoresis (TTGE) for sequence-specific separation. Electrophoresis was performed in 1 mm thick, 16×16 cm polyacrylamide gel (7% wt/vol acrylamide/Bis, 7 M urea, 1.25X TAE, and, respectively, 55 ml and 550 μl of TEMED and 10% ammonium persulfate) using 25 litres of 1.25X TAE as the electrophoresis buffer in a TTGE detection system (CBS Scientific Co, San Diego, CA, USA). The TTGE conditions included electrophoresis at 50 mA for 12 h at an initial temperature of 66°C and a ramp rate of 0.3°C/h. To improve resolution, the voltage was set at 20 mA for 15 min at the beginning of each run. Each well was loaded with 100 to 200 ng of amplified DNA plus an equal volume of 2X gel loading dye (0.05% bromophenol blue, 0.05% xylene cyanol, and 70% glycerol). Gels were stained in the dark by immersion for 15 min in a solution of ethidium bromide stain and were read using the Chemicapture software in a Chemi-Smart imaging system (Vilber Lourmat, Marne-la-Vallee, France). Each sample analyzed in this study was run in duplicate and consensus data compiled. In order to determine variations between PCR and TTGE, DNA samples from a subset of subjects were amplified in duplicate by PCR and TTGE was run again on another day. These samples were compared to each other by TTGE on the same gel, and PCR amplicons were run again on a different day and gel to determine gel-to-gel variation. Composite data sets for group-specific TTGE profiles were generated and numerical band matching character tables produced. The BioDiversity Pro statistical package (version 2; Scottish Association for Marine Science, http://www.sams.ac.uk) was used for principal component analysis. The SDR software (version 4.1.2, Pisces Conservation Ltd., http://www.pisces-conservation.com) was used for diversity analysis.

### Cloning of the PCR products and clone library analysis

Cloning PCR was performed using the same primers but without the GC clamp in the reverse primer. The purified PCR products were cloned into *E. coli* DH5α using the pCR™ 2.1-TOPO^R^ vector (TOPO TA cloning kit, Invitrogen, Carlsbad, CA, USA). Forty five recombinant clones were randomly selected for analysis. The plasmids were purified (Purelink Plasmid Miniprep Kit, Invitrogen, Carlsbad, CA, USA) and were used as templates for amplification of the 239 bp *C. leptum* group-specific 16S rRNA gene fragment with the above mentioned primers without GC clamp for reverse primers. The amplicons were subjected to TTGE analysis. If two clones migrated to the same distance, only one clone was selected for sequencing. All clones which migrated to different distances were selected for sequencing. A total of 23 clones were sequenced using M13 forward primer (Bioserve Biotechnologies, Hyderabad, India). Clones which were sequenced were run on the TTGE analysis along with one sample (individual A) to identify the phylogeny of individual bands on TTGE.

### Sequence analysis

Sequences from our clones and sequences retrieved from the GenBank database were taken to a length of 239 bp corresponding to the *C. leptum* amplicon length. Sequences obtained in our study were aligned with the sequences in the Ribosomal Database Project (release 9) database to determine their closest relatives. Sequences were aligned separately using ClustalX 2.0.9 and analyzed using PHYLIP version 3.68 after 1000 bootstrapping operations. The distance matrices on these datasets were created using DNADIST (PHYLIP version 3.68) and the sequences were subsequently clustered using NEIGHBOUR (PHYLIP version 3.68). The tree files from NEIGHBOUR were applied with CONSENSE (PHYLIP version 3.68) and the consensus tree was viewed using PHYLODRAW.

### Real time PCR assays for *C. leptum* group and *Faecalibacterium prausnitzii*

Real-time quantitative (q) PCR was performed using a Chromo4 four color real time PCR detection system with Opticon Monitor 3 software (Bio-Rad, Hercules, USA). Amplification and detection were carried out in 96-well plates with Mesa Green qPCR Master Mix (Eurogentec SA, Liege, Belgium). Primers were directed at 16S rRNA sequences specific to *C. leptum* and *F*. *prausnitzii*. The primers used to quantify *C. leptum* were the same as those used for TTGE but without the GC clamp in the reverse primer. The primers used to quantify *F. prausnitzii* were as follows: Forward primer 5^′^-GGAGGATTGACCCCTTCAGT-3^′^ and reverse primer 5^′^-CTGGTCCCGAAGAAACACAT-3^′^[19]. Primers were synthesized and purchased from Sigma Aldrich Chemicals (Bangalore, India). Each reaction was done in duplicate in a final volume of 20 μL. Amplifications were performed as follows: 95°C for 5 minutes, to denature DNA and followed by 40 cycles of 94°C for 10 seconds, 58°C for 30 seconds and 72°C for 30 seconds. The final extension step at 72°C for 10 min was followed by a melting curve analysis which was performed by increasing the temperature from 40 to 95°C, with a raise in temperature by 1°C every 10 sec with a plate read step to read the fluorescent signal. DNA copy was expressed as copy number per gram of feces and samples were run in real time PCR along with plasmid standards.

### Statistical analysis

Normally distributed data was compared using ANOVA with post-hoc Bonferroni tests, while data that was not distributed normally was compared using the Kruskal-Wallis test with post-hoc Dunn tests.

## Results

Twenty CD patients (6 female) with mean (SD) age of 31.2 (14.1) years were recruited. They had disease duration ranging from 6 months to three years. Eleven of the patients had a CDAI score of 150 or more, and were categorized as having active CD. Twenty two UC patients (9 female) with mean (SD) age 38.4 (11.3) years were recruited. They had disease duration ranging from six months to eight years. Nine of the UC patients had a UCAI score of 3 or more, and were categorized as having active UC. None of the patients was on treatment with antibiotics. All the IBD patients received treatment with aminosalicylates. Seven of the CD patients and six of the UC patients also received azathioprine. Four of the CD patients had undergone surgery earlier. None of the patients had colonic lavage or colonoscopy within the month prior to stool collection. Seventeen HC (8 female) with mean (SD) age 31.1 (13.8) years were recruited.

### Clone library analysis of *C. leptum* cluster and identification of TTGE bands

A total of 45 clones were obtained which were amplified and run on TTGE. Only clones which differed in migrating position were selected for sequencing. Thus 25 clones were sequenced. The analysis showed that our cloned sequences had high similarity with known sequences available in GenBank. Most of the clones that we isolated belonged to genus *Faecalibacterium* (65%), *Subdoligranulum* (8%) and *Ruminococcus* (4%). The recombinant clones in the library were amplified using primers sg-Clept-F and sg-Clept-GC-R3, and the amplified products were subjected to TTGE in order to generate the identity of each of the TTGE bands.

### Nucleotide sequence accession numbers

The sequences generated in this study were deposited in the GenBank database under accession numbers [GQ465348 to GQ465370].

### TTGE profiles for *C. leptum* group

**Figure 1:**
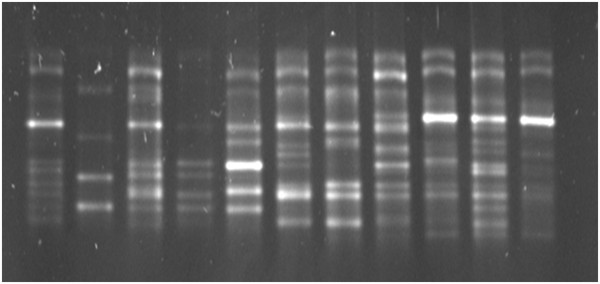
**TTGE gel showing bands migrating at different positions.** Each lane depicts fecal DNA amplified using the Clept primers from an individual participant. Details of the TTGE conditions are provided in the text.

The PCR amplicons were run on TTGE (Figure 1), the image documented and a binary data matrix created by the presence or absence of bands at a specific location. Each band was considered as an operational taxonomic unit (OTU) equivalent to a species. Totally we observed 25 species for the *C. leptum* group by TTGE.

### Multivariate analysis of the TTGE profiles

**Figure 2:**
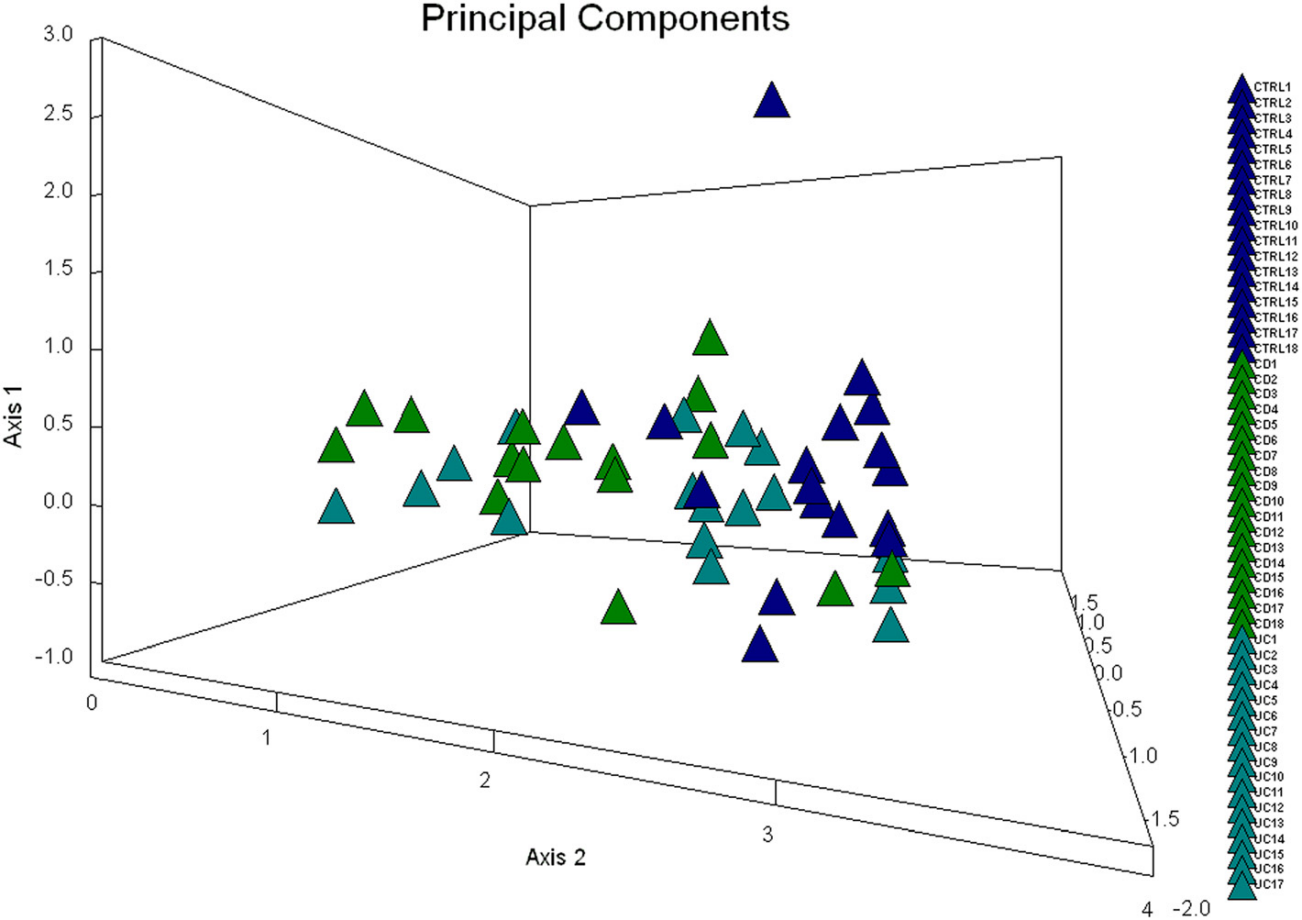
**Principal components analysis of fecal *C. leptum *group bacterial communities. ***C. leptum* species dominantly represented in healthy controls (HC, blue) tend to cluster to the right side of the plot, while those species dominant in Crohn’s disease (CD, green) and ulcerative colitis (UC, turqoise) patients tend to cluster to the left of the plot.

Principal component analysis (PCA) of the TTGE profiles obtained from the healthy subjects and IBD patients demonstrated that the bacterial community profiles were dissimilar by the first and the second principal component (x and y axes on the PCA plot, respectively) (Figure 2). HC were predominantly grouped to the right side of PCA1 whereas CD and UC were more likely to cluster on the left side in PCA1, suggesting that the microbial communities of the healthy individuals shared some characteristics that differentiated them from IBD patients. CD and UC appeared to cluster together in the plot.

### Cluster analysis of the TTGE data

**Figure 3:**
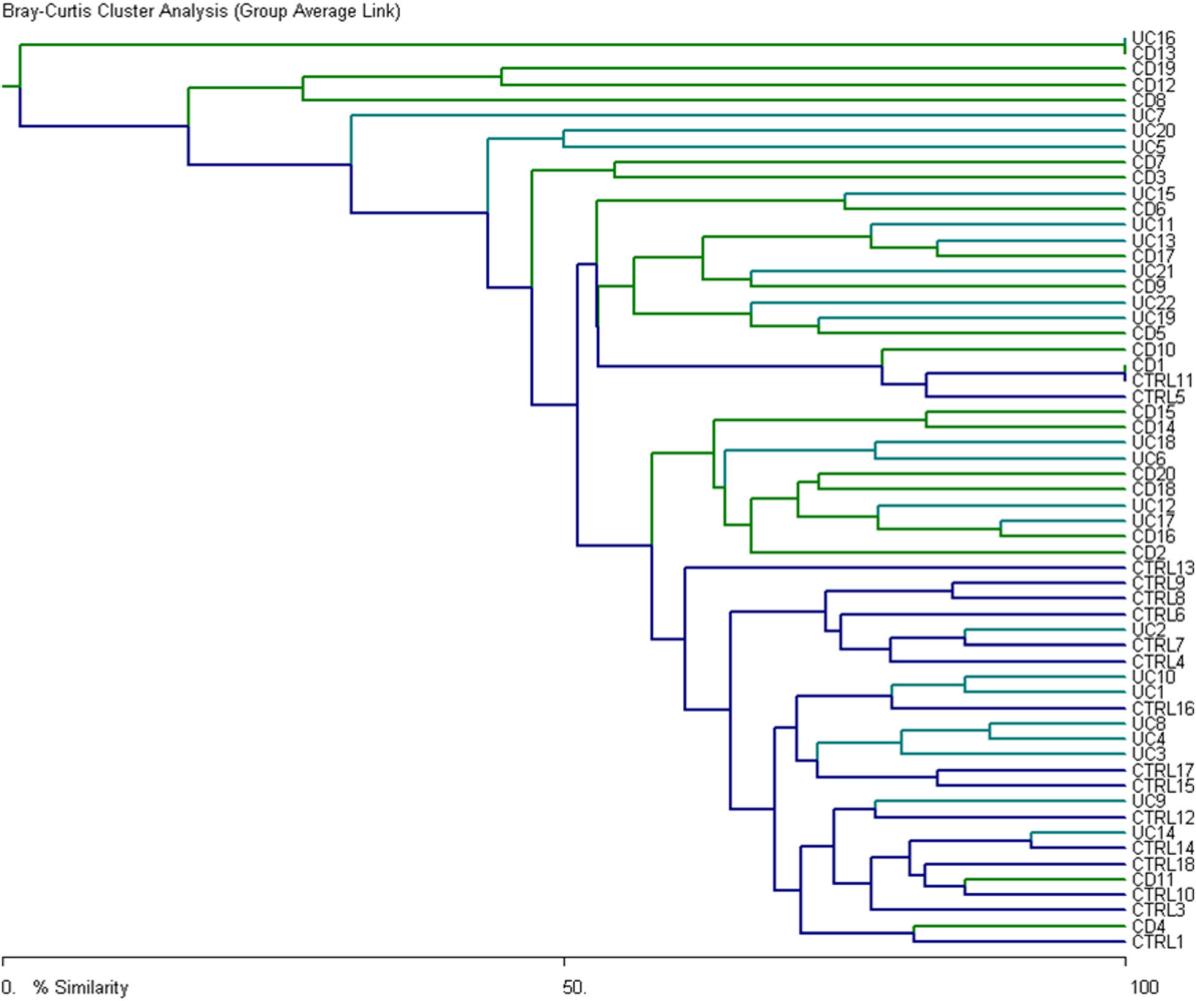
**Bray Curtis cluster analysis of fecal *C. leptum *group communities.** Bacteria from Crohn’s disease (CD) and ulcerative colitis (UC) patients appeared to have higher degrees of dissimilarity to each other than bacteria from healthy controls (HC).

The TTGE binary data, that is the presence or absence of bands, was analyzed by cluster analysis using Bray’s cluster index (Figure 3). In this analysis all sample data were analyzed together, including HC, CD and UC. Compared to the healthy controls, most of the CD and UC patients showed higher dissimilarity in the dendrogram, which again confirmed the observation of PCA. At cut-off 70% below that similarity, controls and IBD groups were clearly differentiated.

### Diversity indices based on TTGE profile

**Figure 4:**
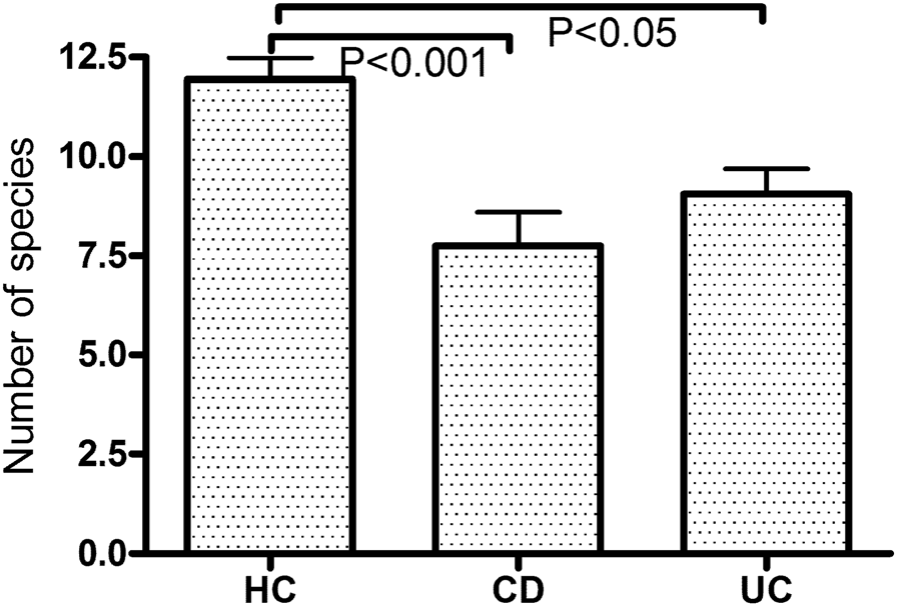
**Number of *C. leptum *species in the feces of individuals in the three groups.** Values shown are mean (SEM).

The number of *C. leptum* group species (Figure 4) was significantly less in CD and UC compared to HC. HC had a mean (SD) of 11.9 (2.2) OTUs in the *C. leptum* group compared to 7.7 (3.7) in CD patients and 9.0 (3.0) in UC patients (P<0.001 HC vs. CD; P<0.05 HC vs. UC; P-NS CD vs. UC). The Simpson’s D index of alpha diversity was 19.65 for HC compared to 17.56 for CD and 17.51 for UC. The difference between HC and CD and UC was not statistically significant.

### Quantitation of *C. leptum* group and *Faecalibacterium prausnitzii* in feces

**Figure 5:**
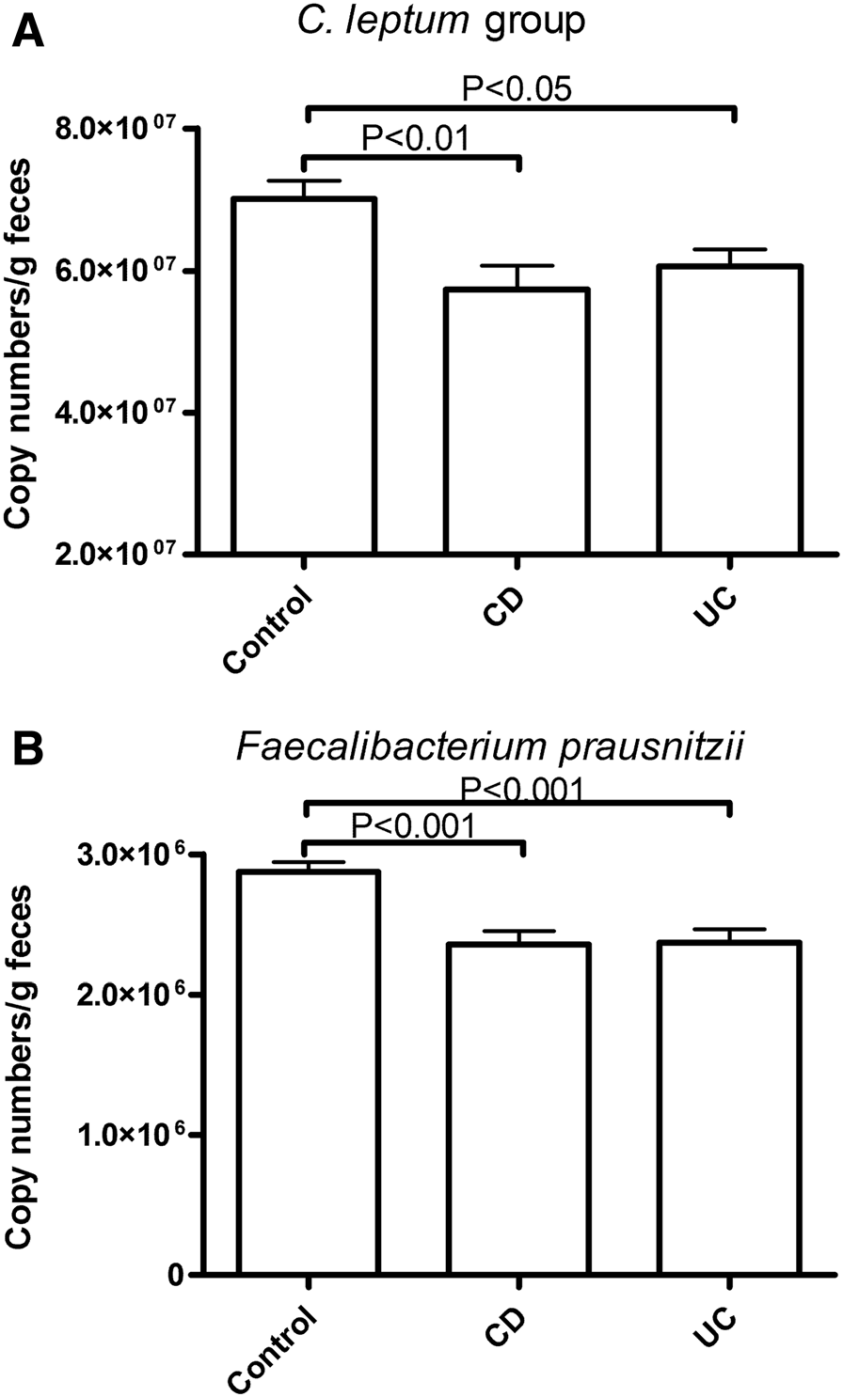
**Abundance of *C. leptum *group bacteria (A) and *F. prausnitzii *(B).** Bacterial abundance in HC, CD and UC, is expressed as mean (SEM) copy number of 16S rRNA gene/g feces.

The count of *Clostridium leptum* group bacteria in feces was significantly reduced in CD and UC compared to healthy controls (HC vs. CD P<0.01; HC vs. UC P<0.05) (Figure 5A). *Faecalibacterium prausnitzii* counts were also significantly reduced in CD and UC patients compared to HC (HC vs. CD P<0.001; healthy vs. UC P<0.001) (Figure 5B).

### Microbiota composition: comparison of active IBD with disease remission

**Figure 6:**
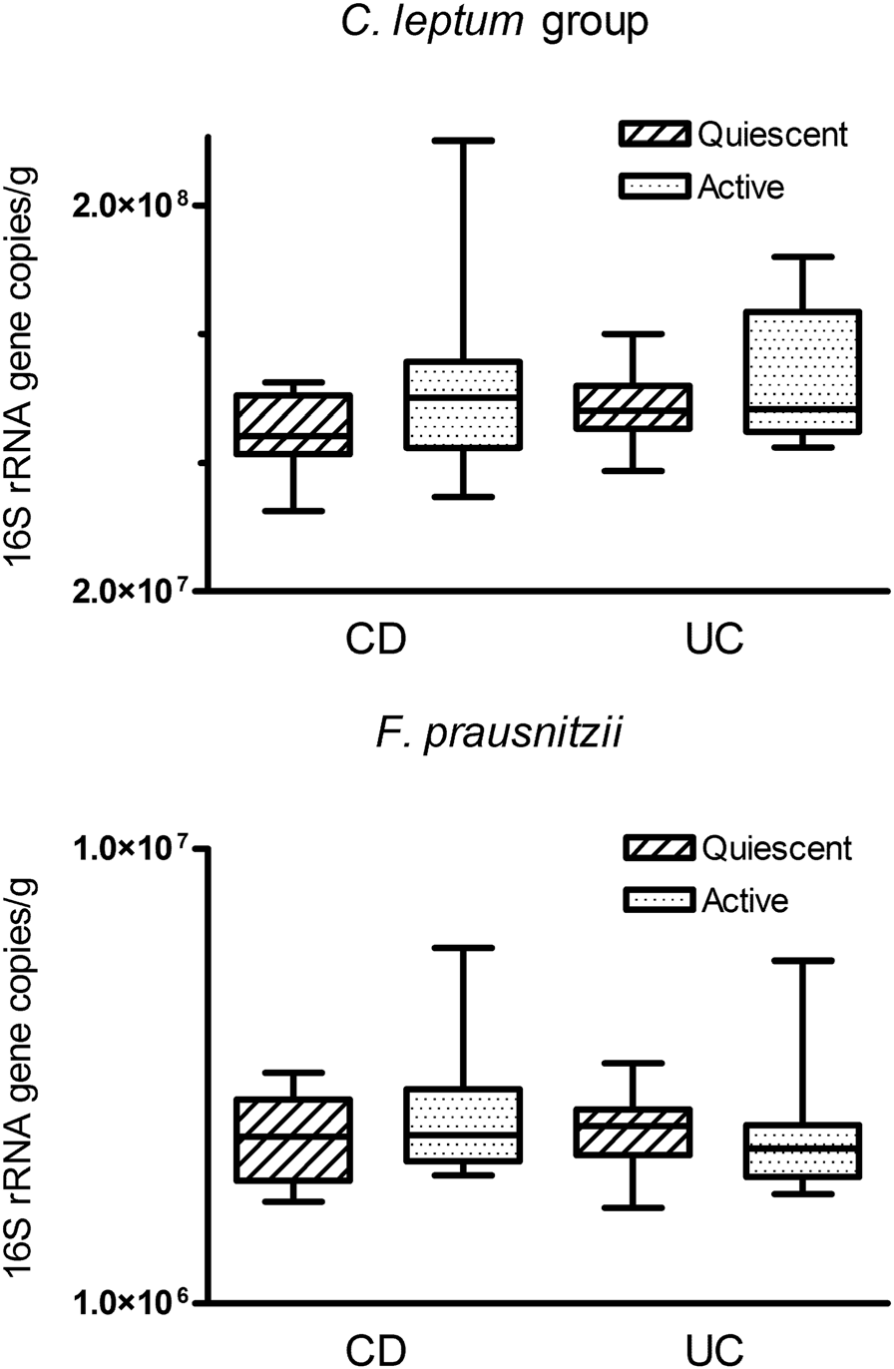
**Abundance of *C. leptum *group bacteria and *F. prausnitzii *in active and quiescent IBD.** Values shown are mean (SEM) of 16S rRNA gene copy number per g feces in patients with CD and UC with quiescent and active disease.

Comparison of *C. leptum* group and *F. prausnitzii* quantitation between IBD patients with active disease (CDAI ≥ 150 or UCAI ≥ 3) and those in clinical remission (CDAI < 150 or UCAI < 3) did not show any quantitative differences between the two groups (Figure 6). There was no correlation of microbial community abundance with duration of diarrhea.

## Discussion

This study, using TTGE to study diversity of *C. leptum* group in IBD patients, provides important information regarding the colonic microbiota in IBD that will augment the existing knowledge in this area. The study shows that there was a reduction of absolute number as well as species number per individual of the *C. leptum* group in both UC and CD. The reduction in numbers was explained to a large extent by the reduction in *F. prausnitzii*, and this alteration was present in both quiescent and active IBD. *C. leptum* group bacteria from IBD patients clustered separately from bacteria present in the healthy controls. Lastly the study provides, for the first time, a catalog of the *C. leptum* group of bacteria in the feces of healthy adult humans in India. The present study focused solely on the *C. leptum* group and *F. prausnitzii* and did not attempt to determine changes in other microbial communities in IBD.

The utilization of culture-independent methods of analysis has significantly enhanced our ability to assess the contribution made by the gut microbiota to human health. In this study, we amplified portions of the 16S rRNA gene that are specific for the *C. leptum* group of bacteria and used TTGE to identify the numbers of species present in in each individual. TTGE is a very sensitive technique to detect single nucleotide changes in DNA. The primer set used is able to detect species of the *C. leptum* group including *C. leptum*, *Eubacterium plautii, Clostridium viride*, and *Ruminococcus albus* strains which are present in small proportions in human fecal samples [19]. TTGE revealed multiple bands, which were cloned and sequenced to reveal at least 25 different species or OTU belonging to this group. The clone library that we generated, as well as the real time PCR data, showed that *F. prausnitzii* was the major constituent (approximately 65%) of this bacterial group. The presence of *Subdoligranulum variabile* as a part of this bacterial group in our participants is consistent with other reports [20].

Healthy controls in the present study had a mean of 12 OTUs of the *C. leptum* group in feces, and this number was significantly reduced in CD and UC patients. However, using the Simpson D index, we did not find any alteration in alpha diversity between HC, UC and CD. Manichanh et al. [14] reported that the species number of *C. leptum*, *C. coccoides*, and certain other Firmicutes was reduced in patients with CD. Their conclusions were based on the analysis of a limited number of clones derived from fecal microbiota of these patients. Other investigators have reported that *C. leptum* diversity was reduced in patients with CD [21]. Principal components analysis showed that *C. leptum* from most of the control subjects clustered discretely to one side whereas *C. leptum* from CD and UC patients clustered separately. This observation needs to be confirmed in more rigorous studies.

Relative abundance of the targeted microbial communities was evaluated using real time PCR in which the specific abundance was expressed relative to amplification of universal bacterial domain sequences of 16S rRNA, i.e. expressed relative to total bacteria. The abundance of *C. leptum* group bacteria, and of *F. prausnitzii*, was significantly reduced in both CD and UC compared to HC. Similar findings have been reported earlier [14,21]. In the present study, there was no significant difference in fecal counts of either *C. leptum* group or *F. prausnitzii* between patients with active IBD and those with quiescent IBD (i.e. those who were in remission). There is little literature on this aspect; however one earlier study suggested that the differences in abundance of *C. leptum* and *F. prausnitzii* occurred in active IBD but not in IBD in remission [9].

The significance of these findings can be interpreted varyingly. *C. leptum* group (cluster IV) is one of the dominant populations of the human fecal microflora [3,22] and contains a large number of butyrate-producing bacteria. It is not known whether the changes in C. leptum number and diversity antedate the development of IBD. However, by showing that changes occur in the same direction in both CD and UC, we can conclude that these changes are not specific to either disease. The alterations were of greater magnitude in CD patients compared to UC patients. We do not have an explanation for this observation. It was also noted in our study that the changes were noted in both active and quiescent IBD, suggesting that the changes were not secondary to gut inflammation and ulceration and presence of pus and blood in the lumen. Probiotics used in IBD are typically a mixture of lactobacilli and bifidobacteria. Species belonging to the *C. leptum* group (esp. *F. prausnitzii*) are also now considered as anti-inflammatory commensal bacteria. Loss of butyrate, which has an anti-inflammatory activity [23], may result in greater inflammation in the colon.

Our data shows decreased diversity of the *C. leptum* group in IBD patients. Increased species diversity is an indication of a robust microsystem. Loss of this diversity is likely to impact on gut health, even if it is not the primary phenomenon driving gut inflammation. The intake of prebiotic substrates like inulin can increase both bifidobacteria and *F. prausnitzii* in the human gut, and this may be one possible intervention to consider in the management of patients with IBD [24]. It is possible that treatment of IBD patients with butyrate producing bacteria such as the *C. leptum* group bacteria will emerge as another therapeutic option in these patients [25].

## Conclusions

Bacteria belonging to the *C. leptum* group, including the dominant constituent *F. prausnitzii*, are significantly reduced in the fecal microbiota of patients with Crohn’s disease and ulcerative colitis. There is a reduction in both the number of species and the absolute number of bacteria in each individual compared to healthy controls. The reduction is found both in active disease and during disease remission, indicating that it is not temporally related to alterations in the intestinal milieu during flares of inflammation. These changes are likely to be significant in the context of the dysbiosis that occurs in patients with IBD.

## Abbreviations

CD: Crohn’s disease; HC: Healthy control; IBD: Inflammatory bowel disease; PCR: Polymerase chain reaction; TTGE: Temporal temperature gradient electrophoresis; UC: Ulcerative colitis.

## Competing interests

None of the authors have a financial or non-financial conflict of interest to declare.

## Authors’ contributions

JK and BSR contributed to study design, data analysis, and write up of the manuscript. JK, VS and SP contributed to laboratory analyses. BSR obtained financial support for these studies. All authors read and approved the final manuscript.

## Pre-publication history

The pre-publication history for this paper can be accessed here:

http://www.biomedcentral.com/1471-230X/13/20/prepub
